# Prospective practice survey of management of cetuximab-related skin reactions

**DOI:** 10.1007/s00520-020-05862-7

**Published:** 2020-11-04

**Authors:** R. T. Lugtenberg, C. B. Boers-Doets, P. O. Witteveen, C. M. L. van Herpen, A. N. M. Wymenga, J. W. B. de Groot, A. Hoeben, C. del Grande, B. van Doorn, J. J. Koldenhof, C. M. L. Driessen, H. Gelderblom

**Affiliations:** 1 Department of Medical Oncology, Leiden University Medical Center, Albinusdreef 2, P.O. Box 9600, 2300 RC Leiden, The Netherlands; 2 Department of Medical Strategy, CancerMed, Wormer, The Netherlands; 3 Department of Adverse Event Research & Valorisation, Impaqtt Foundation, Wormer, The Netherlands; 4 Department of Medical Oncology, University Medical Center Utrecht, Utrecht, The Netherlands; 5 Department of Medical Oncology, Radboud University Nijmegen Medical Center, Nijmegen, The Netherlands; 6 Department of Medical Oncolog, Medical Spectrum Twente, Enschede, The Netherlands; 7 Department of Medical Oncology, Isala Oncology Center, Zwolle, The Netherlands; 8 Department of Medical Oncology, School for Oncology and Developmental Biology (GROW), Maastricht University Medical Center+, Maastricht, The Netherlands; 9 Merck B.V., Netherlands, an affiliate of Merck KgaA, Darmstadt Germany, Schiphol-Rijk, The Netherlands

**Keywords:** Cetuximab, Skin reaction, Management, Head and neck cancer, Colorectal cancer

## Abstract

**Purpose:**

Evidence-based guidelines on how to prevent or treat cetuximab-related skin reactions are lacking and multiple care and management strategies are used. The main purpose of the present study is to gain information about the different skincare products being used against skin reactions in metastatic colorectal cancer (mCRC) and recurrent/metastatic (R/M) or locally advanced (LA) squamous cell cancer of the head and neck (SCCHN) patients treated with cetuximab.

**Methods:**

An open-label, prospective observational study conducted in the Netherlands. The occurrence of skin reactions and the care and management options taken were documented for 16 weeks, starting from the first administration of cetuximab.

**Results:**

A total of 103 patients were included in 7 hospitals. 38 patients (37%) developed a grade ≥ 2 skin reaction. Eighty-six patients could be analysed for the primary endpoint (73.3% males, mean age 62.4 years, *n* = 44 LA SCCHN, *n* = 16 R/M SCCHN, *n* = 26 mCRC). The most frequently used skin products at some point during the observation period were moisturizing products (70%), systemic antibiotics (64%), topical antibiotics (58%), lipid-regenerating (28%) and other topical products (28%). The overall use of products gradually increased from baseline to week 6–10, reducing by week 16. Hospital protocols were the primary reason (> 50%) for choice of the skincare products and medications.

**Conclusion:**

A variety of skin care products and antibiotics were commonly used. Only few patients developed severe cutaneous reactions. For patients, the occurrence of skin reactions did not influence their willingness to continue cetuximab therapy.

## Introduction

Cetuximab is a chimeric monoclonal antibody that binds and inactivates the epidermal growth factor receptor (EGFR). As a consequence of this blockade, extracellular activators like the epidermal growth factor (EGF) cannot bind to the receptor anymore and tumour-promoting downstream signalling cascades cannot be activated. This mechanism of blocking EGFR is an important strategy in the treatment of metastatic colorectal cancer (mCRC) and squamous cell cancer of the head and neck (SCCHN) [1–4].

However, EGFR is expressed not solely on tumour cells but also on cells of the epidermis. There, EGF/EGFR-mediated signalling cascades regulate physiologic homeostasis of the tissue. Inhibition of EGFR causes abnormal growth and migration behaviour of keratinocytes. It also changes keratinocyte differentiation and maturation. These changes are accompanied by an inflammatory state of the dermis [5, 6]. In light of the physiologic relevance of the EGFR signalling cascade, one side effect of this type of drug can be explained: various cutaneous toxicities.

Most patients receiving cetuximab develop skin reactions. In the majority of cases, skin reactions like xerosis, maculo-papular rash, papulo-pustular rash, pruritus or fissures occur within the first weeks of therapy and the occurrence is time-dependent. The most frequently reported events are papulo-pustular rash (80%) which affects predominantly the face, upper trunk and the scalp. Skin reactions caused by cetuximab can severely affect patient health-related quality of life (HRQoL) and can lead eventually to dose delays, dose reductions and even permanent discontinuation of treatment [7, 8]. Normally, skin reactions resolve once the therapy is stopped or following dose reductions. Cetuximab-induced skin reactions have been positively associated with better treatment response and longer survival in mCRC and R/M SCCHN [9–11]. Therefore, finding the optimal strategies to prevent, recognize early and treat skin reactions seems necessary. During cetuximab therapy multiple care and management options to reduce the severity of the skin reactions are used. Secondary to general measures like the avoidance of intense sun exposure and the use of disinfectant synthetic detergents and proper skin care is recommended. However, most recommendations on how to prevent or treat skin reactions are based on expert opinions, since evidence-based guidelines are scarce [12]. Optimal strategies for the management of cetuximab-induced skin reactions remain unclear. Besides the prophylactic use of emollients, use of oral or topical tetracyclines and topical application of 1% hydrocortisone cream together with a moisturizer can be considered to reduce the severity of skin reactions [13, 14], or other topical agents such as vitamin K_1_ cream and antihistamines in case of pruritus [15, 16]. As cetuximab is increasingly being used in cancer therapy, optimal strategy to detect and treat anti-EGFR-induced skin reactions can help clinicians to improve patient care.

The primary objective of this current practice survey is to gain information about the perceived effectiveness of the measures taken against the skin reactions in mCRC and SCCHN patients treated with cetuximab in Dutch daily practice.

## Methods

### Study design

We performed a prospective observational study with a non-experimental cohort design. The primary objective of the study was to provide insight into the use of the different applied prophylactic and reactive skincare products used in patients with cetuximab-related skin reactions. Secondary objectives were incidence and grading of skin reactions as measured by NCI-CTCAE v4.03: reasons for premature discontinuation of cetuximab treatment; reasons for choice of applied skin products; the percentage of days under skin care products during the observation period, HRQoL as measured by the FACT-EGFRI-18; the assessment of the perceived effectiveness of the applied skin products by physicians, nurses and patients.

### Patients

15 to 20 centres within the Netherlands that treat metastatic colorectal cancer (mCRC) and/or recurrent, metastatic or locally advanced squamous cell cancer of head and neck (SCCHN) patients with cetuximab were planned to include 100 patients. Cetuximab treatment consisted of monotherapy or in combination with radiation therapy or chemotherapy. Eligible patients were ≥ 18 years of age, with a histologically proven SCCHN or RAS wild type mCRC who were planned for cetuximab treatment; a wash-out period of 3 months for previous treatment with cetuximab; an Eastern Cooperative Oncology Group (ECOG) performance status ≤ 2; absence of active skin reactions/infections for which use of any topical treatment was needed; absence of the presence of a skin condition in the face, neck or chest that may obscure skin reactions to cetuximab (e.g. excessive facial hair, excessive scarring, sunburn or other disfigurements); and written informed consent.

### Data collection

From the first administration of cetuximab (baseline visit) and during follow-up visits the healthcare professional (treating physician or (research) nurses), using online eCRF, evaluated and assessed skin reactions, skin care management and other patient characteristics. Follow-up visits were scheduled for mCRC and R/M SCCHN patients at weeks 2, 4, 6, 10 and 16, and for LA SCCHN patients at weeks 2, 4, 6 and 8–10, since these patients only receive cetuximab for 8 weeks. Skin reactions were assessed as measured by the National Cancer Institute’s Common Terminology Criteria for Adverse Events (NCI-CTCAE) v4.03. HRQoL was assessed with the FACT-EGFRI-18, a questionnaire developed to assess HRQoL related to dermatologic reactions from EGFRI treatment and were completed by patients before every follow-up visit [17, 18]. The FACT-EGFRI-18 is an 18-item Likert-scaled questionnaire, with response scores ranged from 0 to 4. Product-specific, perceived effectiveness was assessed by using 5 point scales and were used for both skin care products (e.g. moisturizers, lipid-regenerating products, antiseptic products, urea-containing products, vitamin K_1_ cream and others ) and pharmacological agents (topical or oral antibiotics, local anaesthetics antihistamines, topical glucocorticosteroids or others).

### Therapeutic plan

Cetuximab was administered once a week intravenously, in an initial loading dose of 400 mg per m^2^ body surface area, followed by doses of 250 mg per m^2^. Patients received premedication with an antihistamine and in some cases also a corticosteroid.

### Statistical analysis

Patients’ medical records and online electronic case report forms (eCRFs) were used for primary data sources. The full analysis set (FAS) included all enrolled patients who had sufficient documentation of cetuximab treatment and sufficient data for the primary endpoint at least at baseline. The safety analysis set (SAS) included all enrolled patients for whom cetuximab treatment was started.

Descriptive statistical methods were used for all variables (e.g. mean, standard deviation, median, interquartile range for continuous variables and proportions for categorical variables). In addition, 95% confidence intervals were calculated for point estimates (mean, median or proportion). Statistical analysis was performed using Statistical Analysis System Version 9.1.3 (NC, USA).

## Results

### Patients

**Fig. 1:**
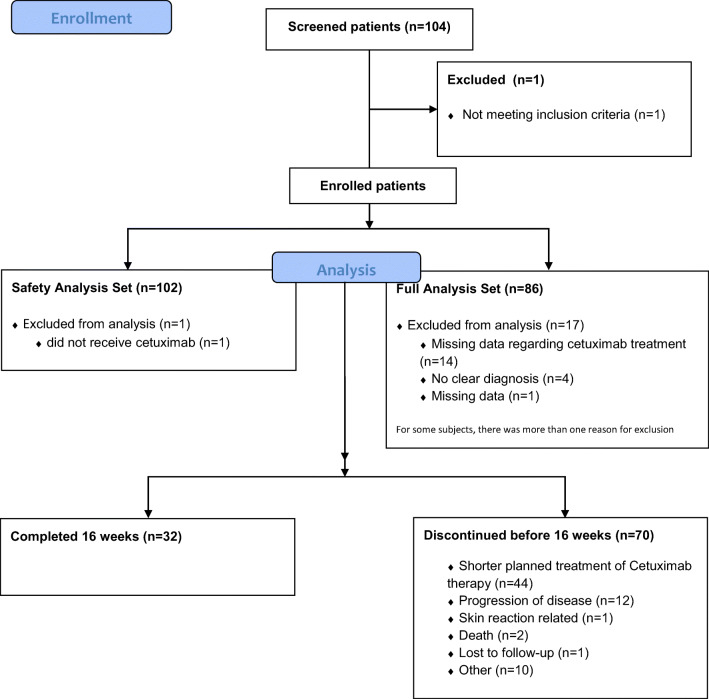
Consort flow diagram

Between September 2013 and December 2016, 103 patients from 7 Dutch centres were enrolled in the study. One of the 103 patients was excluded from the Safety Analysis Set (SAS) because the patient withdrew from the study prior to start of treatment (Fig. 1). Eighty-six out of the 103 patients (83.5%) were included in the full analysis set (FAS). Reasons for 17 patients being excluded from the FAS were missing data: no clear diagnosis of mCRC or R/M SCCHN recorded (*n* = 4); no documented cetuximab treatment (*n* = 14) and insufficient data regarding the primary endpoint at baseline (*n* = 1). For some subjects, there was more than one reason for exclusion. Patients’ demographic characteristics from the FAS are summarized in Table 1. Most patients were male (73%) with a median age of 62 years (range 31–80). Cetuximab was administered in the majority of patients for locally advanced SCCHN (*n* = 44, 51%) as primary treatment, with a planned treatment period of 8 weeks. 26 patients were treated for mCRC (30%) and 16 patients received cetuximab for R/M SCCHN (19%).

**Table 1:** Demographic characteristics and baseline data

Characteristic	(*N* = 86)
Sex, *n* (%)	
Male	63 (73.3)
Female	23 (26.7)
Age (years)	
*n* (%)	86 (100)
Mean ± SD	62.4 ± 10.02
Min; max	31; 80
Body surface area (m^2^)	
*n* (%)	86 (100)
Mean ± SD	1.92 ± 0.24
Min; max	1.43; 2.73
Diagnosis, *n* (%)	
mCRC	26 (30.2%)
R/M SCCHN	16 (18.6%)
LA SCCHN	44 (51.2%)
ECOG performance score, *n* (%)	
0	30 (34.9%)
1	49 (57.0%)
2	7 (8.1%)
Previous anticancer treatment, *n* (%)	
Surgery	22 (25.6)
Chemotherapy	29 (33.7)
Radiation therapy	17 (19.8)
Biological	4 (4.7)
Other treatment	5 (5.8)
No previous anticancer treatment	44 (51.2)
Planned treatment duration, *n* (%)	
Until progression/death	27 (31.4)
Fixed number of cycles	55 (64.0)
Missing	4 (4.7)
Planned treatment duration (weeks)	*N* = 51
Mean ± SD	7.7 ± 3.0
Min; max	1; 24
Line of treatment, *n* (%)	
Primary	44 (51.1)
1st line	15 (17.4)
2nd line	14 (16.3)
3rd line	11 (12.8)
Later	1 (1.2)
Missing	1 (1.2)

*SD*, standard deviation; *mCRC*, metastatic colorectal cancer; *R/M*, SCCHN metastatic or recurrent squamous cell cancer of the head and neck; *LA SCCHN*, locally advanced squamous cell cancer of the head and neck; *ECOG*, Eastern Cooperative Oncology Group

### Skin reactions incidence and grading

89 of 102 patients (87%) developed a skin reaction at some point during the observation period. Table 2 lists the incidence and severity of the skin reactions that occurred. The majority of these reactions were grade 1 or 2 (45 and 37% respectively). Xerosis was reported in 68 patients (67%), maculo-papular rash in 55 (54%), papulo-pustular rash or acneiform eruption in 54 (53%) and pruritus in 34 (33%) patients. Less often observed reactions were paronychia, hand-foot skin reaction and acneiform rash.

**Table 2:** Skin reactions by most severe grading in the safety analysis set (*n* = 102)

Most severe grading	Total, *n* (%)	Grade 1, *n* (%)	Grade 2, *n* (%)	Grade 3, *n* (%)	Grade 4, *n* (%)	Unknown, *n* (%)
Any skin reaction	89 (87.3)	46 (45.1)	38 (37.3)	5 (4.9)	0	0
Rash maculo-papular	55 (53.9)	39 (38.2)	12 (11.8)	2 (2.0)	0	2 (2.0)
Rash papulo-pustular	54 (52.9)	30 (29.4)	20 (19.6)	4 (3.9)	0	0
Xerosis	68 (66.7)	53 (52.0)	12 (11.8)	0	0	3 (2.9)
Pruritus	34 (33.3)	27 (26.5)	7 (6.9)	0	0	0
Paronychia	10 (9.8)	8 (7.8)	2 (2.0)	0	0	0
Hand-foot skin reactions	5 (4.9)	3 (2.9)	2 (2.0)	0	0	0
Other skin reactions	40 (39.2)	30 (29.4)	7 (6.9)	1 (1.0)	0	2 (2.0)

### Premature discontinuations

70 of 102 patients (69%) discontinued participation in this study before 16 weeks. Main reasons were a planned treatment period of cetuximab treatment of 8 weeks in patients with locally advanced SCCHN (44 patients); disease progression (12 patients); death (2 patients); lost to follow-up (1 patient); or other reasons (10 patients). Only one patient discontinued prematurely due to skin reactions.

### Skin products used

A summary of the use of the different applied skin care products and medications administered to prevent or treat skin reactions is provided per visit in Table 3. The results of week 16 reflect on the patients who were still treated with cetuximab at that timepoint and were therefore mainly mCRC patients. At baseline, the most frequently used skin care products were moisturizing products (38%), systemic antibiotics (20%), lipid-regenerating products (12%), vitamin K_1_ cream (9%) and topical antibiotics (6%). None of the patients used topical steroids, antiseptic products or urea-containing products at baseline. During the observation period of the study, the most frequently used skin products were moisturizing products (70%), systemic antibiotics (64%), topical antibiotics (58%), lipid-regenerating products (28%) and other topical treatments (28%). The overall use of products showed a gradual increase from baseline to week 6/10 for most of the products, reducing by week 16. The overall use of topical antibiotics increased from baseline (6%) to week 6 (51%) reducing by week 16 (31%). The overall use of systemic antibiotics increased from baseline (20%) to week 4 (48%) reducing by week 16 (28%). The overall use of other topical treatments gradually increased from week 2 (8%) to week 10 (23%) reducing by week 16 (10%). The overall use of moisturizing products increased from baseline (38 %) to week 6 (51%) reducing by week 16 (35%). The overall use of lipid-generating products was identical at baseline and week 2 (12%) and increased by week 6 (26%) reducing gradually by week 16 (3%). The overall use of vitamin K_1_ cream increased from baseline (9%) to week 4 (48%) reducing by week 16 (28%). Other medications and skin care products like antihistamines, antiseptic and urea-containing products were used in less than 15% of patients. Wet wraps were not used.

**Table 3:** Overall use of the different skin care products

Medication/skin care products	Baseline (*N* = 86)	Week 2 (*N* = 86)	Week 4 (*N* = 82)	Week 6 (*N* = 78)	Week 10 (*N* = 56)	Week 16 (*N* = 29)
Topical antibiotics						
*n* (%)	5 (5.8)	24 (27.9)	39 (47.6)	40 (51.3)	25 (44.6)	9 (31.0)
95% CI	1.9–13.0	18.8–38.6	36.4–58.9	39.7–62.8	31.3–58.5	15.3–50.8
Systemic antibiotics						
*n* (%)	17 (19.8)	28 (32.6)	39 (47.6)	35 (44.9)	26 (46.4)	8 (27.6)
95% CI	12.0–29.8	22.8–43.5	36.4–58.9	33.6–56.6	33.0–60.3	12.7–47.2
Moisturizing product						
*n* (%)	33 (38.4)	36 (41.9)	41 (50.0)	40 (51.3)	25 (44.6)	10 (34.5)
95% CI	28.1–49.5	31.3–53.0	38.7–61.3	39.7–62.8	31.3–58.5	17.9–54.3
Lipid-regenerating product						
*n* (%)	10 (11.6)	10 (11.6)	18 (22.0)	20 (25.6)	6 (10.7)	1 (3.4)
95% CI	5.7–20.3	5.7–20.3	13.6–32.5	16.4–36.8	4.0–21.9	0.1–17.8
Vitamin K_1_ cream						
*n* (%)	8 (9.3)	12 (14.0)	11 (13.4)	9 (11.5)	8 (14.3)	5 (17.2)
95% CI	4.1–17.5	7.4–23.1	6.9–22.7	5.4–20.8	6.4–26.2	5.8–35.8
Other topical treatments						
*n* (%)	0	7 (8.1)	14 (17.1)	16 (20.5)	13 (23.2)	3 (10.3)
95% CI		3.3–16.1	9.7–27.0	12.2–31.2	13.0–36.4	2.2–27.4

*CI*, confidence interval

### Reasons for the choice of the applied skin care products

The hospital protocol was the primary reason for choice of the skin care products at baseline (58%) and during treatment (up to 71%). Remaining reasons were investigator choice, previous experience, advice by a dermatologist or other reasons (< 20% each).

### Percentage of days under skin care products

The proportion of days on skin treatment during the observation period (from the day of the first cetuximab treatment until 2–4 weeks after the last administration of cetuximab) was in a similar range for the specific type of treatments (about 50 to 70%).

### Health-related quality of life

A continuous decrease in HRQoL, as measured by the FACT-EGFRI-18, was seen during the observation period, with the strongest deterioration at week 10 followed by a slight improvement at week 16. Both the total and all domain scores of the FACT-EGFRI-18 decreased over time, indicating that the treatment that patients received in this study, consisting of cetuximab monotherapy, or combined with radiation therapy or chemotherapy, had effect on physical functioning and overall well-being.

### Perceived effectiveness of applied skin products

**Fig. 2:**
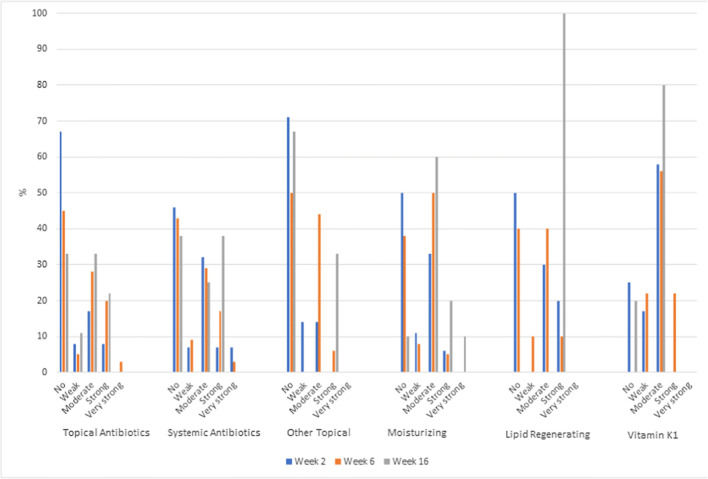
Healthcare providers’ perceived effectiveness of applied skin products

For almost all products effectiveness ratings varied across patients and HCPs from “no effect”, “weak” and “moderate/strong”, while “very strong” was rarely mentioned. During the observation period for all products, an increase in the percentage of patients who perceived at least weak efficacy was observed. Moderate to strong effectiveness was perceived most often by patients using moisturizing products (up to 90% at week 16); vitamin K cream (up to 78% at week 6); systemic antibiotics (up to 63% at week 16); topical antibiotics (up to 50% in weeks 6-16); and lipid-regenerating products (up to 50% from week 2 and beyond). The HCPs’ perceived effectiveness of the applied skin products is summarized in Fig. 2. In general, the percentages of the response “no effect” declined while “moderate” gained percentages over time.

### Mean HCPs’ perceived effectiveness of different agents on skin reactions across visits

The average numerical effectiveness values (from 0 = no, to 4 = very strong) of the assessments across all visits for each patient and type of medication were analysed with calculation of the mean ± standard deviation (SD). Mean perceived effectiveness by HCPs (regardless of prophylactic or reactive usage) was highest for vitamin K_1_ cream (1.32 ± 0.736 in 14 patients), lipid-regenerating product (1.05 ± 0.752 in 24 patients) and systemic antibiotics (1.05 ± 1.066 in 55 patients). The treatments with the lowest values were topical antibiotics (0.96 ± 0.925 in 50 patients), moisturizing products (0.92 ± 0.826 in 60 patients) and other topical treatments (0.85 ± 0.887 in 24 patients). The perceived effectiveness of prophylactic treatment was 0.90 ± 1.025 for moisturizing products in 37 patients and 0.26 ± 0.554 for systemic antibiotics in 16 patients. The results in the reactive group hardly differed with mean perceived effectiveness ranging from 0.67 ± 0.900 to 1.14 ± 1.232 for the different skin products.

### Patients’ impression of skin reactions

The vast majority of patients reported “neutral” impact of skin reactions on daily life during the total study period (95.3% in week 2, 96.2% in week 6 and 93.1% in week 16). Only 2 patients reported no impact of skin reactions on daily life. None of the patients reported “very strong” impact on daily life. Skin reactions did not influence the willingness to continue cetuximab therapy in most patients (97.7% in week 2, 93.6% in week 6 and 93.1% in week 16). At week 2, 1 patient (1.2%) strongly favoured continuation of therapy, increasing to 4 patients (5.1%) in week 6. Furthermore, there was one patient (1.2%) at week 2 who strongly favoured discontinuation of therapy.

**Fig. 3:**
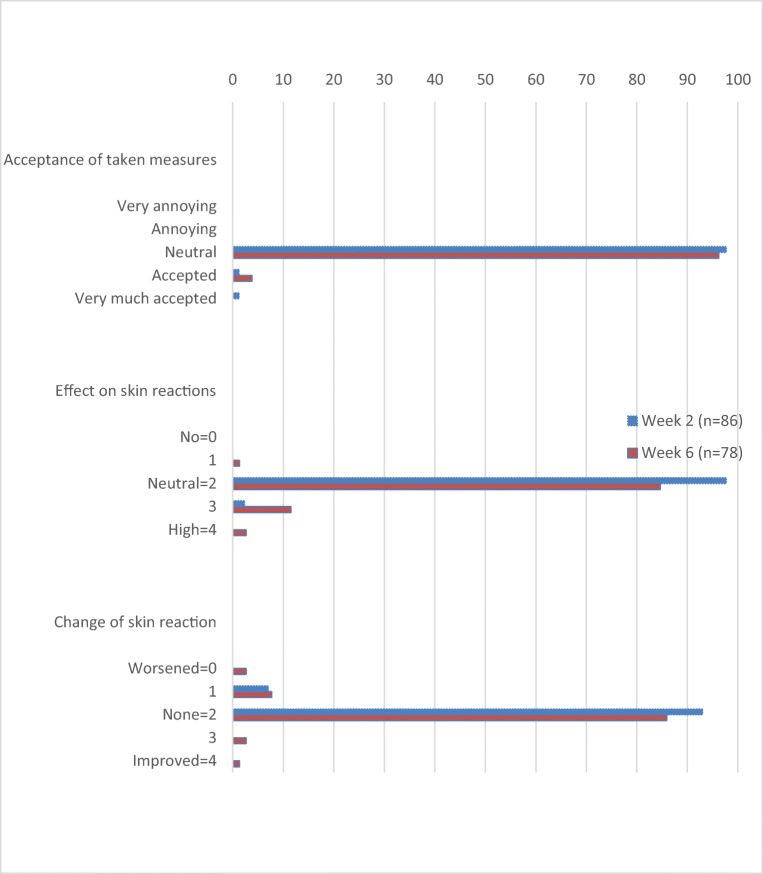
Patients’ perceptions of the measures taken and effect on skin reactions

Patients’ perceptions of the measures taken are summarized in Fig. 3. The majority of the patients who reported the perceived measures taken against skin reactions to be neutral reported a neutral effect of skin products on their skin reactions and no change in the skin reactions during the observation period.

## Discussion

This prospective observational study provides a detailed insight into the use, acceptance and the perceived efficacy of measures taken against skin reactions related to cetuximab in the Netherlands. A wide range of skincare products and medications was used in patients with metastatic colorectal cancer (mCRC) and recurrent and/or metastatic or locally advanced squamous cell cancer of head and neck (SCCHN) in 7 sites across the country. The most frequent skin products in the study were moisturizing products, systemic antibiotics and topical antibiotics wherein the use of all products gradually increased during the first 10 weeks of the observation period. Hospital protocols were the primary reason for the choice of skin care and medical products being used as prophylactic or reactive treatment of skin reactions.

These findings are comparable with the results of a similar study conducted in Switzerland, in which patients also received a broad variety of preventive and therapeutic skin care measures for cetuximab-related skin reactions [19]. In the absence of evidence-based guidelines for an optimal approach to prevent and manage cutaneous reactions of EGFR-targeted antibodies, our practice survey and the Swiss study demonstrate the differences in choice of skin care products in the various hospitals and areas. Only a small proportion of patients developed severe cutaneous reactions and for patients, the occurrence of skin reactions did not influence their willingness to continue cetuximab therapy. However, the two studies differ in some ways. In contrast to the Swiss study, the minority of patients in our study was diagnosed with mCRC (30.2% versus 72.8%) and patients were allowed to undergo radiation therapy at the same time. In addition, there was a difference in the choice of skin care products. General skin care products frequently used in Switzerland like urea-containing products, lipid-regenerating products and vitamin K_1_ cream were rarely prescribed in the Dutch population. On the other hand, antibiotics (topical and systemic) were more popular in the Netherlands. This can partly be explained by the fact that vitamin K_1_ cream was not available by prescription in the Netherlands when the study was conducted. In addition, hospital protocols have often been drawn up in consultation with dermatologists, whereas differences in dermatological care between countries are common. Despite these differences in patients and chosen skincare products, the outcome in the studies was in accordance. Remarkably, topical steroids were rarely described in both countries, despite the fact that these agents are often recommended in expert guidelines.

This study was conducted to gain more information about skin reactions related to cetuximab therapy and current measures taken to prevent and manage them in daily practice. It should be mentioned that only 26 out of 102 patients (25.5%) received monotherapy with cetuximab. 48 patients (47.1%) had concurrent radiation therapy, 27 patients (26.5%) received a combination of chemotherapy and cetuximab and 1 patient (1.0%) was treated with chemotherapy, palliative radiotherapy and cetuximab at the same time. Therefore, not all skin reactions are all necessarily cetuximab-related. There might have been patients with radiation dermatitis or a chemotherapy-induced rash as well. Up to 85 to 95% of patients treated with radiotherapy developed moderate to severe skin reactions depending on the cumulative radiation dose to the skin [20–22]. Optimal strategies for the prevention and treatment of radiation-induced dermatitis remain a challenge as well with conflicting results in various studies. Washing with water and mild soap, topical corticosteroids and silver nylon dressings have proven to be effective in radiation dermatitis; all strategies were not applied in this survey.

Cutaneous reactions caused by cetuximab can have a serious impact on patients’ HRQoL and their willingness to continue treatment [23]. In the present study, no grade 4 skin reactions were observed and less than 5% of patients had a grade 3 reaction at some point during the observation period. This has ensured that only 1 patient has stopped treatment with cetuximab due to skin reactions. We did see a decrease in the mean HRQoL overall score, as for all domains, from baseline to week 10. Possibly, mild or moderate skin reactions can negatively affect patients’ HRQoL and daily life. However, because the majority of patients received radiation or chemotherapy, the declined HRQoL scores cannot only be attributed to cetuximab-related skin reactions. The fact that a large proportion of patients have only been treated for 8 weeks may also have helped the treatment tolerance, as patients can experience a greater negative impact if side effects last longer.

Patients, physicians and nurses perceived the effectiveness of the used products generally comparable for all categories. “No effect” or at most “weak to moderate” effectiveness was reported by the majority of participating patients and physicians. A proportion of patients used multiple products at the same time, like moisturizing products and systemic antibiotics, which made it difficult to distinguish which products were less effective. Patients seemed to be most enthusiastic about moisturizing products, vitamin K_1_ cream and antibiotics (systemic and topical), whereas healthcare providers reported the highest scores for vitamin K_1_ cream, lipid-regenerating products and systemic antibiotics. The differences between perceived effectiveness assessments were small with no superiority for one of the used agents. In concordance with our Swiss colleagues, we have to conclude that the optimal treatment option needs to be explored individually [19]. Results from previous studies, recommendations and discussions at consensus meetings remain conflicting and so far, no evidence-based guideline for cetuximab-related skin reactions could be established.

There are a number of limitations to this study. Firstly, as a consequence of missing data only 86 patients could be analysed resulting in a relatively small sample size. It was the intention to analyse all endpoints from mCRC and SCCHN patients separately; however, the low number of patients in each subgroup and the high number of skin care medications given precluding meaningful interpretation of data in the subgroups. In addition, it would have been useful to compare the incidence, time of occurrence and grade of skin reactions in patients given prophylactic treatment and those not receiving such treatment. Due to a lack of sufficient data regarding the type of treatment given, this analysis could not be performed in a meaningful way. Secondly, the follow-up period of 16 weeks is relatively short, and many patients discontinued the study early as they were treated for a shorter planned period with cetuximab. Although the development of skin reactions in patients treated with cetuximab usually starts within the first weeks of treatment. Therefore, this time window was chosen for the observation period in this study. Thirdly, the present results were potentially biased in some aspects. Inclusion of patients in a non-blinded observational study with no control arm, at which the treating HCP assigns patients to a certain therapy of their own preference, might have influenced the reported perceived effectiveness. Furthermore, the inclusion of only Dutch patients with access to all available highly qualified care and a good health insurance system might limit the generalizability of these findings to patients in lower- or middle-income countries.

## Conclusions

Our study shows that Dutch oncologists and nurses used a variety of products for the prevention or treatment of cetuximab-induced skin reaction, mostly based on the local hospital protocols. In general, both patients and treating healthcare providers perceived at most moderate efficacy of the various measures. Fortunately, the skin reactions that occurred during treatment were mostly mild or moderate, had no large impact on daily life and did not lead to discontinuation of treatment.

## Data Availability

The datasets generated and/or analysed during the current study are not publicly available as sharing is not explicitly covered by patient consent.
